# Intravital imaging of metastasis in adult Zebrafish

**DOI:** 10.1186/s12885-017-3647-0

**Published:** 2017-09-25

**Authors:** David C. Benjamin, Richard O. Hynes

**Affiliations:** 1 Department of Biology, Massachusetts Institute of Technology, 31 Ames Street, Cambridge, MA 02139 USA; 2 David H. Koch Institute For Integrative Cancer Research, Massachusetts Institute of Technology, 500 Main Street, Cambridge, MA 02139 USA; 3 Howard Hughes Medical Institute, 4000 Jones Bridge Road, Chevy Chase, MD 20815 USA

**Keywords:** Zebrafish, Metastasis, Intravital imaging

## Abstract

**Background:**

Metastasis is a major clinical problem whose biology is not yet fully understood. This lack of understanding is especially true for the events at the metastatic site, which include arrest, extravasation, and growth into macrometastases. Intravital imaging is a powerful technique that has shown great promise in increasing our understanding of these events. To date, most intravital imaging studies have been performed in mice, which has limited its adoption. Zebrafish are also a common system for the intravital imaging of metastasis. However, as imaging in embryos is technically simpler, relatively few studies have used adult zebrafish to study metastasis and none have followed individual cells at the metastatic site over time. The aim of this study was to demonstrate that adult *casper* zebrafish offer a convenient model system for performing intravital imaging of the metastatic site over time with single-cell resolution.

**Methods:**

ZMEL1 zebrafish melanoma cells were injected into 6 to 10-week-old *casper* fish using an intravenous injection protocol. Because *casper* fish are transparent even as adults, they could be imaged without surgical intervention. Individual cells were followed over the course of 2 weeks as they arrested, extravasated, and formed macroscopic metastases.

**Results:**

Our injection method reliably delivered cells into circulation and led to the formation of tumors in multiple organs. Cells in the skin and sub-dermal muscle could be imaged at high resolution over 2 weeks using confocal microscopy. Arrest was visualized and determined to be primarily due to size restriction. Following arrest, extravasation was seen to occur between 1 and 6 days post-injection. Once outside of the vasculature, cells were observed migrating as well as forming protrusions.

**Conclusions:**

*Casper* fish are a useful model for studying the events at the metastatic site using intravital imaging. The protocols described in this study are relatively simple. Combined with the reasonably low cost of zebrafish, they offer to increase access to intravital imaging.

**Electronic supplementary material:**

The online version of this article (10.1186/s12885-017-3647-0) contains supplementary material, which is available to authorized users.

## Background

Metastasis is the cause of the overwhelming majority of cancer-related deaths, yet our understanding of the underlying biology remains incomplete [1]. The events that occur at the metastatic site (namely arrest, extravasation, and growth into a new tumor) are particularly poorly understood [2]. These events are in need of further elucidation because they may be rate-limiting steps in the metastatic cascade, as evidenced by the fact that tumors can shed thousands of cells per day into circulation yet only a small fraction of these will go on to form metastases [3]. Studies of events at the metastatic site have indicated that dynamic interactions between tumor cells, platelets [4, 5], leukocytes [6, 7], and endothelial cells [8] are key in regulating the formation of metastases. These interactions have been challenging to study in mice due to their transient nature and occurrence deep within vital organs [9]. The development of intravital imaging techniques has begun to allow the observation of these events.

The current state of the art for intravital imaging in mice involves the surgical implantation of glass windows through which a tissue of interest can be imaged [10]. Protocols have been developed for imaging common sites of metastasis including the lung [11], liver [12], brain [13], and bone marrow [14]. Once these windows have been installed, the animal can be imaged repeatedly over multiple weeks. These techniques have been used to study tumor cell arrest [13], interactions with immune cells [15, 16], and their early outgrowth into metastases [13, 17].

While imaging windows have allowed the study of metastatic sites in living mice, some key limitations have restricted their widespread adoption. First, each animal in an experiment requires the surgical implantation of an imaging window. The time required to prepare each animal limits the number that can be used in an experiment. Second, the equipment, expertise and time required to become proficient in the surgical techniques make intravital imaging a far-from-routine technique. Finally, certain tissues present further technical challenges beyond the installation of an imaging window. For example, the lung is one of the best-studied metastatic sites in mice [18]. However, imaging the lung requires additional stabilization techniques to compensate for respiratory movements which reduce the duration of imaging [19]. These techniques can involve the ex vivo isolation of the lung [20] or methods to adhere the lung to imaging windows in vivo [11, 21] to minimize its movement. The additional challenges associated with these techniques have limited the number of intravital imaging studies in murine lungs.

Zebrafish embryos have been extensively used as a model system for the intravital imaging of metastasis [22–24] owing to their optical transparency and development outside the mother. In addition, zebrafish share a great deal of homology with humans, with approximately 70% of human genes having an identifiable homolog in zebrafish [25]. However, it remains unclear how well the embryonic microenvironment and remodeling vasculature recapitulate the situation in an adult fish.

The recent development of a transparent line of zebrafish, *casper* [26], offers an adult model for the intravital imaging of cancer and metastasis. *Casper* fish carry two homozygous mutations that prevent the development of melanophores and iridophores. Without these two types of pigment cells, zebrafish are transparent even as adults, eliminating the need for any further manipulation of the animal prior to experimentation. *Casper* fish have been used to image the clonal heterogeneity [27, 28] and neovascularization [27] of transplanted primary tumors. *Casper* fish have also been used as a quantitative system to study metastasis using fluorescence as a readout [29]. In addition, micrometastases have been detected in tumor-bearing *casper* fish following the transplantation of tumors [27]. However, the events at the metastatic site have not been studied in adult *casper* fish.

We describe here a protocol for the intravenous injection of tumor cells into young adult *casper* fish that is an improvement on current methods used for adult injections. We then describe a simple protocol for intravital imaging and demonstrate its utility by characterizing the behavior of tumor cells at the metastatic site over the course of two weeks.

## Methods

### Zebrafish husbandry

Zebrafish were housed in a room maintained at 28 °C with a 14-h light, 10-h dark cycle. Fish not in experiments were housed in a re-circulating water system and fed brine shrimp three times a day. During experiments, fish injected with zebrafish tumor cells were housed individually in plastic cups containing approximately 400 mL of aquarium makeup water (AMW) and were fed brine shrimp once per day. Zebrafish injected with human tumor cells were maintained in glass bottles in 100 mL of AMW in a 34 °C water bath and fed brine shrimp once per day. Prior to experimentation, zebrafish were acclimated to the increased temperature by raising the water temperature by 1 °C per day.

The *casper* (*roy* ^*−/−*^ *;nacre* ^*−/−*^ *)* line was a kind gift from Dr. Leonard Zon (Boston Children’s Hospital). The *flk:dsRed2* line was originally developed in the laboratory of Dr. Kenneth Poss (Duke) and was a kind gift from Dr. Mehmet Yanik (MIT). It was crossed into the *casper* background. The *rag2* ^*450fs/+*^ and *prkdc* ^*D3612fs/+*^;*casper* lines were kind gifts from Dr. David Langenau (MGH). The *rag2* ^*450fs*^ line was crossed into the *casper* background. The *rag2* ^*450fs/+*^ *;casper* and *prkdc* ^*D3612fs/+*^ *;casper* lines were maintained as heterozygotes. Heterozygotes were crossed, and homozygotes, which were used in experiments, were identified using previously described genotyping protocols [28, 30]. Young adult zebrafish used for experiments were between 6 and 10 weeks old and were housed at a density of 15 fish per 3 L tank. Fish that were noticeably smaller than the majority of the fish in a tank were not injected. Prior to injection with ZMEL1 zebrafish melanoma cells [29], immunocompetent *casper* fish were irradiated with two doses of 15 Gray (Gy), one and two days prior to injection, using a GC 40E gamma irradiator (Theratronics).

Prior to injection with human tumor cells, zebrafish were either irradiated with 15Gy or 20Gy at one and two days before injection, or treated with 15 μg/mL dexamethasone starting 2 days before injection. Embryos were maintained at 34C for the course of experiments involving human cells. Embryos were dechorionated by adding 12uL of 30 mg/mL pronase (Sigma) to a 10 cm dish containing 80 embryos 16 h prior to injections.

### Histology

Zebrafish were euthanized by soaking in 0.1% tricaine on ice for 20 min. Fish were then fixed for 24 h in Bouin’s fixative (Sigma). Following fixation, fish were soaked in water for 3 h and decalcified by soaking in Richard Allan decalcifying solution (ThermoFisher) for 16 h. Fish were then rinsed with water, dehydrated in ethanol, embedded in paraffin, and cut into 5 μm thick sections. Sections were stained with hematoxylin and eosin (H&E) using a ThermoShandon Varistain Gemini (ThermoFisher) staining machine according to manufacturer’s instructions.

### Cell culture

The ZMEL1 zebrafish melanoma cell line [29] was maintained in DMEM high-glucose medium (ThermoFisher) supplemented with 10% fetal bovine serum (FBS, Sigma), L-glutamine (2 mM, ThermoFisher), and primocin (0.1 mg/mL, Invivogen) as were the human breast cancer line, LM2 (A kind gift from Dr. Joan Massagué (Memorial Sloan Kettering Cancer Center)), and the human melanoma cell line, MA2 (previously described [31] ATCC CRL-3223). MDA-MB-435 human melanoma cells (ATCC HTB-129) were cultured in L15 medium (Thermofisher) supplemented with 10% FBS, L-glutamine (2 mM, ThermoFisher), primocin (0.1 mg/mL, Invivogen), bovine insulin (0.01 mg/mL, Sigma), and glutathione (0.01 mg/mL, Sigma). ZMEL1 cells were grown at 32C with 5% CO_2_. LM2 and MA2 cells were grown at 37C with 5% supplemental CO_2_. MDA-MB-435 cells were grown at 37C without supplemental CO_2_.

### Intravenous, retro-orbital, and embryo injections

Cells for injection were harvested from a confluent 10 cm plate by trypsinization for 5 min with 2 mL of 0.25% trypsin in versene. Trypsin was quenched using 4 mL of serum-containing medium. Cells were washed twice with sterile phosphate-buffered saline (PBS) and were re-suspended at 5 × 10^5^ cells/μL in sterile PBS (intravenous), 1 × 10^4^ cells/μL in sterile PBS (retro-orbital), or 4 × 10^6^ cells in 100μL sterile PBS (embryos).

For retro-orbital injections, a removable needle syringe (Hamilton) with a 26-gauge 15 mm length needle with point style 4 and a 30-degree angle (Hamilton) was used. 1 μL of cell suspension was injected retro-orbitally into each fish as previously described [32]. The needle was rinsed with 70% ethanol and PBS between each injection.

For intravenous and embryo injections, glass capillary tubes (Borosilicate, 1 mm outer diameter, 0.58 mm inner diameter, Warner Instruments) were siliconized using Sigmacote reagent (Sigma). Briefly, both ends of the tubes were dipped in Sigmacote until the reagent filled the entire tube. Sigmacote was removed from tubes by dipping tubes onto a Kimwipe (Kimtech) and allowing reagent to flow out. Tubes were then submerged in distilled, deionized water to remove HCl by-products. The water was removed and the capillaries were allowed to dry for 24 h.

Siliconized glass capillary needles were pulled on a P-87 micropipette puller (Sutter Instrument) set to heat = 800, pull = 150, vel =150, time = 200 and pressure = 600. Pulled needles were then used on a picospritzer II (General Valve) microinjector set to dispense 30 ms pulses at 25PSI. The dispensed volume was measured using an ocular micrometer (Nikon) by dispensing 0.5% phenol red solution into mineral oil. The needle tip was progressively broken until it dispensed 20 nL drops for intravenous injections or 2 nL for embryo injections. Once filled, the needle was used to inject into the common cardinal vein of 8 adult fish before being refilled. The needle was only changed if it broke during the course of injections. Multiple fish were anesthetized and injected in rapid succession to minimize the time for cells in suspension to settle in the needle.

Prior to intravenous injections, young adult zebrafish were anesthetized in 0.017% tricaine (Sigma) in AMW buffered to pH 7.4 with sodium bicarbonate. Fish were allowed to remain in anesthetic-containing water until unresponsive to touch. Once anesthetized, fish were transferred to a dry 10 cm dish cover for injection. Following injection, fish were transferred to AMW without anesthetic to recover.

Embryos were anesthetized in 0.017% tricaine in AMW buffered to pH 7.4. Once all swimming ceased, 30 embryos were placed into a 10 cm dish half-filled with 2% agarose and the water was removed. 4 nL of cells in PBS were injected into the Duct of Cuvier where it enters the heart as previously described [22]. Embryos were then washed off with fresh AMW, and housed in 24 well plates (one fish per well) for the duration of the experiment.

### Confocal Intravital imaging

Anesthesia was induced by placing a single fish in 50 ppm eugenol (Pulpdent Corp) in AMW. Once opercular movements slowed, the fish was placed in one well of a 6-well glass bottom dish (Mattek, uncoated 1.5 glass thickness) such that the posterior was in the center of the glass and the head rested on the plastic surrounding it. The posterior of the fish was then immobilized by adding 2% low-melt agarose dropwise. A small amount of 15 ppm eugenol in AMW was added dropwise to the gills to allow respiration while the agarose solidified. Once the agarose solidified, the well was filled with 15 ppm eugenol in AMW. Fish were monitored every minute for opercular movements. If they ceased, fresh AMW without eugenol was added until they resumed. If the fish began to wake up, 50 ppm eugenol in AMW was added for 2 min to re-induce anesthesia and was then replaced with 15 ppm eugenol for maintenance of anesthesia. Fish were imaged on a Nikon-A1R inverted confocal microscope. Images for quantification were acquired using the resonant scanner. Representative images were acquired with the galvanometer scanner. Overview fields to identify vascular landmarks and quantify cell numbers were taken using a 10× objective to image a Z-stack containing 17 steps with a 6.8 μm step size. Extravasation and cell morphology was observed using a water immersion 40X long-working-distance objective to obtain a Z-stack of 49 steps with a 0.3 μm step size.

Representative images were processed in ImageJ (NIH) following acquisition. Briefly, images were first de-speckled and contrast-adjusted. Images were then stacked using the Z-projection function with the maximum-intensity algorithm. Contrast was further adjusted using the shadows and highlights function in Photoshop (Adobe).

### Imaging on a dissecting microscope

Prior to imaging, zebrafish were anesthetized in 0.017% tricaine in AMW buffered to pH 7.4 with sodium bicarbonate. Zebrafish were then placed on a dry 10 cm dish top cover. The area around the fish was dried, leaving a small meniscus of water covering the fish to allow for respiration. Fish were briefly imaged using a Leica M165 FC dissecting microscope.

### Embryo imaging

Each well of a 96 well glass-bottom plate (MatTek) was filled with 60μL of 2% agarose. A 3D–printed pin tool was placed into the wells to generate molds for holding the embryos in a lateral position as previously described [33]. Embryos were anesthetized during imaging in 0.017% tricaine in AMW buffered to pH 7.4. Embryos were imaged on a Nikon A1R inverted confocal microscope. Embryos were imaged with a 10X objective to collect Z-stacks with 14 steps and a 7.4μm step size.

## Results

### Development of adult intravenous injections

**Fig. 1:**
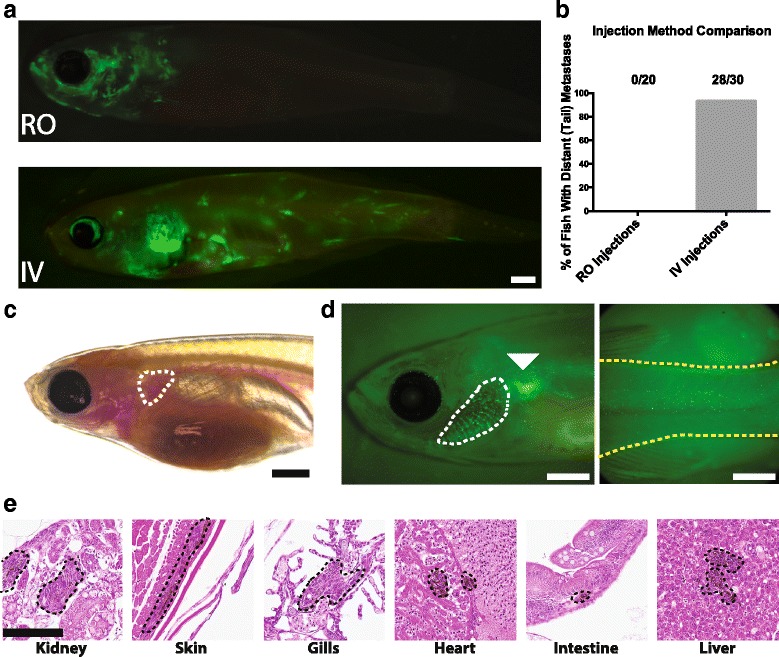
Intravenous injection of zebrafish melanoma cells (ZMEL1) into adult zebrafish. **a** Representative images of zebrafish injected with GFP-labeled zebrafish ZMEL1 melanoma cells 14 days after retro-orbital (*top*) and intravenous (*bottom*) injections. Scale bar is 1 mm. **b** Quantification of injection efficiency of retro-orbital and intravenous injections as determined by the presence of distant metastases in the posterior of the fish with the success rate indicated. **c** 6 to 10-week-old *casper* fish with injection location (common cardinal vein) outlined. Scale bar is 1 mm. **d** Example of a successful intravenous injection as indicated by GFP-labeled tumor cells in the gills (*white dashed line*) and posterior of a *casper* fish (*yellow dashed line*) 1 h post-injection. The injection site is indicated with a white arrowhead. Scale bar is 1 mm. **e** H&E stained transverse sections of zebrafish 14 days post-injection showing tumors in the indicated organs. Tumors are indicated by *black dotted lines*. Scale bar is 100 μm

Retro-orbital (RO) injections are a common route for injecting cells or reagents directly into circulation in adult zebrafish [32]. However, in our hands, these injections have a low efficiency rate in young adult fish (6 to 10-weeks-old) as determined by the percentage of fish with tumors in the posterior of the animal 14 days post-injection (Fig. 1a and b). The posterior of the animal was chosen for quantification as all injections led to a tumor at the injection site, in the anterior of the animal, but only successful injections disseminated cells to the posterior.

In transparent *casper* fish, the major blood vessels are visible through the skin, allowing direct intravenous (IV) injections. We chose to inject into the common cardinal vein because it is a large target that is easy to locate under a dissecting microscope (Fig. 1c and Additional file 1A). Intravenous injections into the common cardinal vein offered improved efficiency compared to retro-orbital injections in 6 to 10-week-old *casper* fish (Fig. 1a and b). We developed the injection protocol using a GFP-labeled zebrafish melanoma cell line (ZMEL1) that has been previously described [29]. This cell line was chosen as it is one of the few zebrafish cancer cell lines available and its metastatic behavior has been well characterized.

Injection success could be determined immediately following injection by observing GFP-positive cells in the gills and posterior of the fish (Fig. 1d). By 14 days post-injection, tumors were observed growing throughout the fish (Fig. 1a). Histology of fish at this time point revealed tumors in multiple organs including the kidney, skin, gills, heart, intestine, and liver (Fig. 1e).

### Intravital imaging of adult zebrafish

**Fig. 2:**
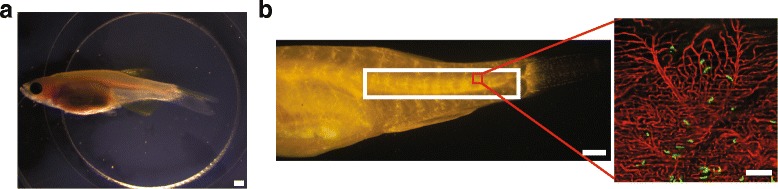
Live imaging of adult zebrafish following injection of ZMEL1 melanoma cells. **a** Intravital imaging set-up with an adult zebrafish restrained in low-melt agarose in a glass-bottomed 6-well plate. Scale bar is 1 mm. **b** Region of imaging in the posterior of a *casper*;*flk:dsRed* zebrafish (*white box*) and example of a single 20× confocal field (*red box*). Scale bar for the posterior is 1 mm. Scale bar for the 20× field is 100 μm

In mice, intravital imaging requires surgical techniques to gain access to the organs of interest [10]. In contrast, *casper* fish require no surgical intervention prior to imaging. We were able to perform intravital imaging in these fish by simply placing them in a glass-bottom dish and immobilizing them with anesthetic and low-melt agarose (Fig. 2a). *Flk:dsRed2* fish were crossed into the *casper* background to allow visualization of the vasculature and tumor cells in living fish. The vasculature in these fish was clearly visible to a depth of 100 μm with confocal microscopy or 200 μm using 2-photon microscopy (data not shown).

The optimal imaging location was determined to be the region just lateral to the spine in the posterior of the fish near the tail fin (Fig. 2b). This region was relatively flat which allowed a large area to be visible in a single focal plane. In addition, this region was far enough from the head to be minimally affected by the fish’s opercular movements. ZMEL1 cells could be observed in the vasculature in the skin and sub-dermal musculature in this region shortly following injection (Fig. 2b). In the first few hours following injection, tumor cells were observed flowing through blood vessels (Additional file 2: Movie S1) or stably arrested (Additional file 3: Movie S2).

### Studies of the early events at the metastatic site

**Fig. 3:**
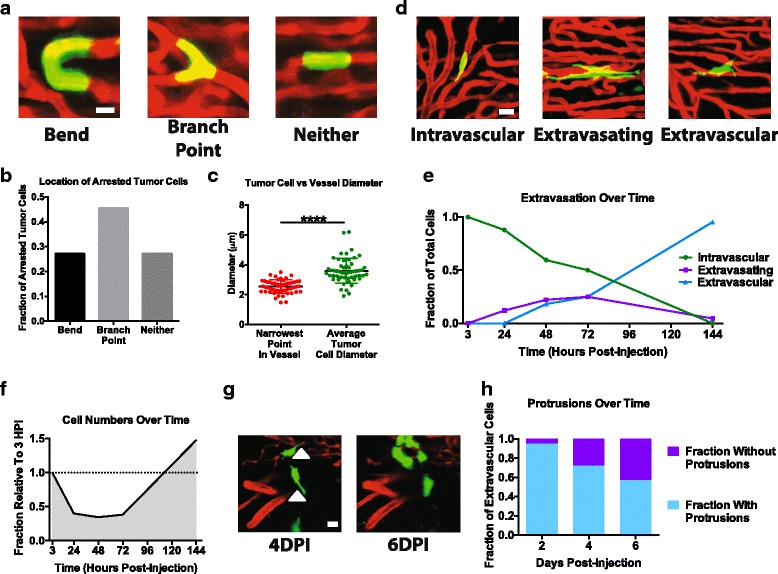
Imaging the early events at the metastatic site. **a** Example images of ZMEL1 zebrafish melanoma cells arrested at bends, branch points, or neither within 3 h of injection into *casper*;*flk:dsRed* fish. Scale bar is 10 μm. **b** Quantification of the fraction of ZMEL1 cells arrested at bends, branch points, or neither within three hours of injection. Quantifications are representative of 170 cells in 8 fish. **c** Quantifications of the diameter of arrested ZMEL1 cells and the diameter of the vessel in which they are arrested within 3 h of injection. *n* = 53 cell and vessel pairs across 5 different fish. *p* < 0.0001 using a two-tailed Student’s t test. **d** Example images of intravascular, extravasating, and extravascular cells 2 days post-injection. Scale bar is 10 μm. **f** Quantification of the fraction of zebrafish melanoma cells that are intravascular, extravasating, and extravascular. Data are representative of 141 cells imaged across 8 different fish. **f** Quantification of the fraction of ZMEL1 melanoma cells remaining over time following injection. Data are representative of 58 fields in 10 fish. **g** Image of ZMEL1 cells 4 and 6 days post-injection showing the loss of protrusions (*white arrowheads*). Scale bar is 10 μm. **h** Quantification of the fraction of ZMEL1 cells with protrusions over time. Data are representative of 164 cells in 3 fish

We next investigated the early events at the metastatic site (namely arrest, extravasation, and early outgrowth of metastases) using intravital imaging. We first characterized the locations where ZMEL1 cells arrested within the first hour following injection. We observed that tumor cells arrested at three categories of locations: bends, branch points, or neither of the two (Fig. 3a). When we compared the relative frequencies of cells at these three locations, we found a majority (73%) of tumor cells arrested at bends and branch points, and the remaining 27% arrested at neither (Fig. 3b). The diameter of tumor cells arrested in vessels was then measured and was found to be larger than the narrowest point in the vessel adjacent to the arrested tumor cell (Fig. 3c). This result suggests that tumor cells in this system arrest once they become lodged in vessels too small for them to travel through.

Following injection with ZMEL1 cells, individual fish were imaged at the time points indicated (Fig. 3e). The non-invasive imaging protocol allowed each fish to be repeatedly imaged without any apparent ill effects. Extravasation was quantified during this time window by scoring cells as intravascular, extravasating, or extravascular (Fig. 3d). Extravasation began at 24 h post-injection and increased until a peak at 72 h post-injection. By 6 days post-injection (DPI), all remaining cells had extravasated or were in the process of extravasating (Fig. 3e).

The attrition of tumor cells at the metastatic site was followed at the same time points as above. Cell numbers were quantified as the fraction of cells relative to the first time point (3 h post-injection). Cell numbers declined initially, reaching a plateau at 48 h post-injection, after which cell numbers steadily increased until the end of the experiment (Fig. 3f). During these imaging studies, we observed that many extravascular cells made long protrusions following extravasation (Fig. 3g). Over time these protrusions were observed to be lost (Fig. 3h).

Following our experiments with the ZMEL1 cell line, we expanded our studies to human tumor cells with well-studied in vivo metastatic phenotypes: the MDA-MB-435 melanoma cell line, the LM2 triple-negative breast cancer cell line, and the MA2 melanoma cell line. Following injection, some cells were observed to have extravasated by 24 h post-injection (Additional file 4A). However, when the same location was imaged over time, MDA-MB-435 cells (data not shown) and LM2 cells were observed to disappear within 2 days of injection (Additional file 4B and C). We suspected that these cells were lost due to clearance by the immune system.

We tested various immunosuppression regimes using irradiation, dexamethasone, or a combination of both based on previous studies that have reported success in establishing tumor xenografts in zebrafish [34, 35]. We also tested two lines of genetically immunocompromised fish: *rag2* ^*450fs/450fs*^ and *prkdc* ^*D3612fs/D3612fs*^.

However, our efforts were ultimately unsuccessful (Additional file 5: Table S1). It is possible that other methods or cell lines would have been successful as other groups have reported success with different cell lines [35, 36], zebrafish lines [37], and immunotolerization approaches [38]. In addition, the continued development of lines of immunocompromised zebrafish suggests that this limitation may be temporary [39].

It is possible that some of these results could be explained by the inability of these human cell lines to grow in zebrafish. While the MDA-MB-435 cells have previously been shown to grow in immunocompromised 4-week-old zebrafish [34], LM2 human breast cancer and MA2 human melanoma cells have not. To assay the growth of the LM2 and MA2 cells in zebrafish, these cell lines were injected into 2-day-old embryos. As the embryo lacks an adaptive immune system, a failure to grow in this context could be the result of an intrinsic inability to grow in zebrafish rather than immune rejection. MA2 metastases in the tail grew between 1 and 4 days post-injection (Additional file 4D). However, LM2 cells were lost during this time period (Additional file 4D). While these results may indicate that the LM2 cells are being lost due to their inability to grow in zebrafish, the fact that MDA-MB-435 and MA2 cells also fail to engraft in adults suggests that immune rejection remains the primary hurdle for successful engraftment.

### Studies of metastases over time

Currently, it is technically challenging to study the growth of metastases from single cells to large metastases in a living organism. We followed ZMEL1 cells over the course of 2 weeks as they formed metastases. We chose 2 weeks as the end-point for these studies because ZMEL1 tumors grow rapidly and it becomes difficult to distinguish individual metastases after this time point. Metastases will continue to grow out until 4 weeks post-injection, by which time the fish begin to look unhealthy and must be sacrificed in accordance with animal welfare guidelines (data not shown).

**Fig. 4:**
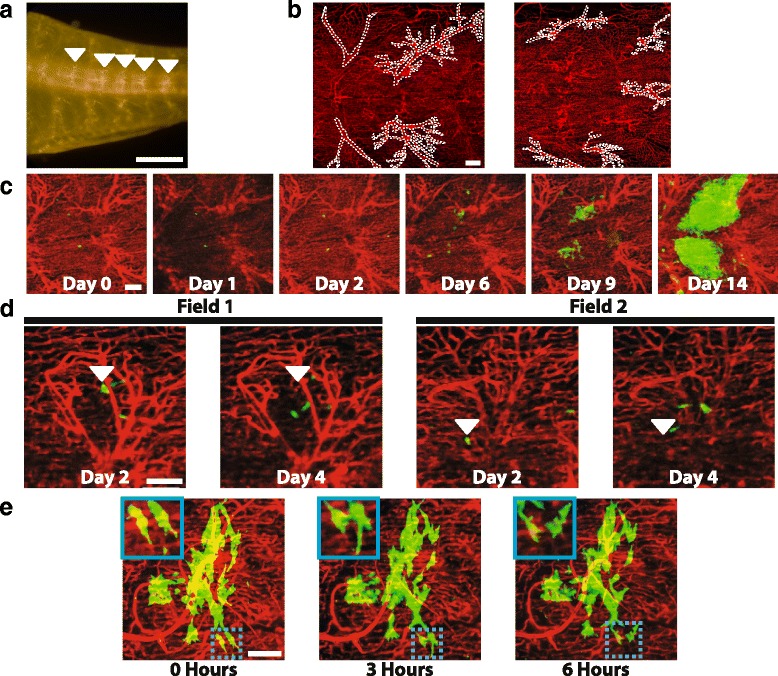
Imaging of disseminated tumor cells over the course of two weeks. **a** Image of the tail of a *flk:dsRed*;*casper* fish with the large vessels used as landmarks indicated by *white arrowheads*. Scale bar is 1 mm. **b** Example 10× fields with the large vessels used for landmarks (*white dotted lines*) highlighted to indicate that the vessels in each field are unique. Scale bar is 100 μm. **c** One field containing ZMEL1 tumor cells imaged over the course of two weeks showing the growth pattern of metastases. Scale bar is 100 μm. **d** Images of two sites on day 2 and day 4 post-injection showing that individual cells are rarely found in the same location during this time period. *Arrowheads* indicate the position of selected cells 2 days post-injection. Scale bar is 100 μm. **e** One field 9 days post-injection that was imaged over the course of 6 h showing cells extending and retracting protrusions. Inset shows higher magnification of two cells that change shape extensively during the 6 h of imaging. Scale bar is 100 μm

In order to return to the same locations over time, the vasculature was used to provide landmarks. In the region of the fish where imaging was performed, large vessels are seen at regular intervals between muscle segments (Fig. 4a). These vessels form unique patterns that can be recorded and later referenced for navigation. Using these vascular patterns (Fig. 4b), we were able to return to the same spot in the fish over 2 weeks.

Regions of the fish containing arrested tumor cells were identified shortly after injection. These regions were then imaged at the time points indicated and single, disseminated tumor cells were followed as they grew into large metastases (Fig. 4c). Following extravasation, cells were quite migratory. Between day 2 and day 4 post-injection, cells rarely remained in the same location (Fig. 4d). Additionally, the morphology of metastases at day 9 post-injection was reminiscent of “pre-micrometastases” observed in early murine liver metastases using intravital imaging [17]. In these “pre-micrometastases,” cells were observed to be highly migratory over the course of 6 h. To test whether the ZMEL1 tumors at day 9 in the zebrafish were also migratory, 9-day post-injection metastases were imaged every 3 h over the course of 6 h. During this time interval, minimal migration was observed. However, cells extensively changed shape by extending and retracting protrusions (Fig. 4e).

## Discussion

In this study, we report techniques for the intravenous injection of tumor cells into young adult zebrafish, as well as an intravital imaging protocol to follow these cells over time as they form metastases. Metastasis is a complex, dynamic process that involves the interactions between tumor cells and many different cell types and factors in the microenvironment [2, 40]. This complexity is extremely difficult to recapitulate in vitro, frequently requiring studies of metastasis to be performed in a live organism, usually a mouse.

Intravital imaging techniques in mice have greatly increased our understanding of these events and the biology underlying them. However, intravital imaging protocols are not trivial, limiting their adoption as a standard laboratory technique. Zebrafish are another a useful model for imaging metastasis in real time in vivo. The majority of these experiments have used 2-day-old zebrafish embryos [22, 23, 41]. However, it is unclear how similar the microenvironment and vasculature in the embryo are to those in adult fish. Adult fish have been used to study invasion and intravasation from a primary tumor but not the latter events of the metastatic cascade [34]. We have shown here that adult *casper* zebrafish offer a useful model system for performing intravital imaging studies of the latter steps of the metastatic cascade.

We first demonstrated the reliable delivery of tumor cells throughout the animal following intravenous injections. Histology 2 weeks post-injection showed delivery to a wide range of organs. This result suggests that this intravenous injection technique could be used to study metastasis to organs besides the skin and muscle, which were chosen in this study for their ease of imaging. Indeed, while we could not image deep into *casper* fish with confocal microscopy, the fluorescent signal was visible on a dissecting microscope and could be used to assay metastatic burden, potentially allowing zebrafish to be used for rapid, cheap experimental metastasis assays.

Following injection, cells could be imaged traveling in circulation and arresting in the vasculature in the first few hours following injection. The most common method for studying these events has been to sacrifice mice at short time points post-injection and analyze fixed sections [5, 6, 42]. While these studies have advanced our knowledge considerably, they have rarely been able follow a single cell over time, so these results are summaries of bulk populations. A few intravital imaging studies in mice have followed single cells over extended time periods [13, 17]. However, there remains a relative paucity of studies that have monitored the behavior of individual cells over time. Of particular interest would be to track tumor cells following interactions with other cell types and observe the influence of these interactions on those cells’ metastatic behavior. A recently developed technique allows for the continuous imaging of an anesthetized zebrafish for up to 24 h [43, 44]. Combining this technique with our injection method could allow for the study of these events with high temporal resolution.

In addition, the relative contribution of specific adhesion molecules and passive mechanical trapping to arrest remains an area of active research [2]. Currently, it remains challenging to perform intravital imaging on more than a few mice in a day. Given that our methods allow the imaging of larger numbers of fish in a day, it would be possible to screen the contributions of multiple adhesion molecules in vivo for their effect on arrest.

Extravasation is similarly a process in which live imaging could provide insight. Currently, most in vivo imaging studies of extravasation utilize embryonic systems (either zebrafish embryos or the chicken chorioallantoic membrane) as they provide easy imaging platforms [22, 45]. It remains unclear how well the remodeling vasculature of an embryo, and the microenvironment in these systems general, recapitulate those of an adult organism. The methods described here can bridge this gap by providing an ease of imaging similar to embryonic systems combined with an adult microenvironment.

The events between extravasation and the emergence of a clinically detectable metastasis remain one of the least well understood events of the metastatic cascade [46]. To date, there have been only a handful of studies that have used intravital imaging to tackle this question [13, 17]. The methods presented here can be used to study the events in this time window. We demonstrated this by following single ZMEL1 cells for 2 weeks following injection. In the first 4 days following extravasation, cells were highly motile. In mice, extravasated tumor cells were also observed to be motile [13, 17] and it has been found that pharmacologic inhibition of this motility can inhibit metastatic outgrowth [17]. These results suggest that a better understanding of motility at the metastatic site would be valuable.

In our experiments, micrometastases 9 days post-injection resembled a previously reported early metastatic phase in the murine liver [17]. However, unlike the situation in the murine liver, we did not observe rapid migration in the sub-dermal musculature on a similar time scale. Instead, the cells were relatively stationary while continuously extending and retracting protrusions. It would be interesting to see the effect of pharmacological inhibition of this activity on metastatic outgrowth.

We attempted to follow human cells in our system as well but found that they were quickly lost, presumably because of clearance by the immune system. We based our immunosuppression regime on two studies that reported success in growing human cells in adult zebrafish [34, 35]. Using these studies as a guide, we tested irradiation, treatment with dexamethasone or both in 6 to 10-week-old fish or in younger 4-week-old fish. As the adaptive immune system is still developing in 4 week old fish, it seemed possible that intervention at this time point might be more effective than at a later stage when the immune system is already fully developed [47]. However, we were unsuccessful in establishing stably growing mammalian tumors.

One possible explanation is that some cell lines are better able to grow in adult zebrafish than others. For example, mouse glioma cells have been reported to grow in adult fish using only dexamethasone immunosuppression [35]. In another study, DU145, K562, and HepG2 cells were all successfully engrafted using only a single dose of 20Gy of irradiation [37]. However, these experiments were performed using a different line of transparent fish (*nacre:rose*) making a direct comparison difficult. The MDA-MB-435 cell line, which we used, has been reported to grow in zebrafish immunosuppressed solely with dexamethasone [34]. We also tested whether the LM2 and MA2 cell lines were able to grow in zebrafish embryos as a test of their ability to grow in a fish environment. While the LM2 cells failed to grow even in the embryo, the MA2 cells did grow over the course of 4 days. These data potentially indicate that immune clearance (rather than a failure to grow in the fish environment) is the more likely explanation for most of our results.

One piece of evidence further supporting this hypothesis is that the strongest combination of dexamethasone and irradiation did allow some tumor growth before the fish died. To try to solve the problem of immune clearance, we tested two lines of genetically immunocompromised fish. The *rag2* ^*450fs/450fs*^ line of zebrafish contains a frame-shift mutation near the C-terminus of the *rag2* gene resulting in a hypomorphic allele. Fish homozygous for this allele lack T cells but still have B cells [30]. These residual B cells could be responsible for the observed rejection of human tumor cells in these fish. The *prkdc* ^*D3612fs/D3612fs*^ fish lacks both B and T cells. In addition, there is another *prkdc* mutant line of fish that was developed around the same time [36]. While the *prkdc* ^*D3612fs/D3612fs*^ line that we used was reported to reject mammalian tumor cells [28], this other *prkdc* ^*−/−*^ line was reported to allow the engraftment of multiple human tumor cell lines [36]. It remains unclear how to reconcile these two conflicting studies. It is possible that differences in the genetic backgrounds or in the *prkdc* mutations themselves are responsible for these differing results.

All of the fish lines mentioned above still have functioning NK cells, which can reject xenotransplanted tumor cells. In mice, it is common to use animals that, in addition to a homozygous *prkdc* mutation, are homozygous for a mutation in the IL2 receptor gamma chain. This mutation serves to eliminate NK cells. Given the rapid development of immunocompromised zebrafish, it seems likely that such fish will soon be available. These or other genetically immunocompromised lines of zebrafish may soon provide the appropriate background for xenotransplantation experiments.

Another approach to deal with rejection by the immune system is to tolerize fish to human tumor cells prior to transplantation. One such study injected irradiated tumor cells into 2-day-old *casper* embryos [38]. The embryos developed normally and these cells were slowly lost over time. However, when adults were later challenged with un-irradiated cells of the same line, tolerized fish developed tumors while naïve fish did not. If this technique can be replicated, it may offer a way around the challenges reported here, albeit, with increased technical complexity.

The above studies show that there is a great deal of heterogeneity in approaches and results of xenotransplantation in adult zebrafish. Recently, allotransplantation has developed into a mature and reproducible technique in adult zebrafish [28–30]. This development coincides with the development of a great number of zebrafish tumor models [48]. Cell lines could be derived from these models, which could then be used with the experimental techniques described here. Working with zebrafish tumor cells also has the advantage of avoiding species incompatibilities that could affect studies of a tumor cell’s interactions with its microenvironment.

## Conclusions

The events at the metastatic site are currently poorly understood. While intravital imaging studies have begun to improve our understanding of them, intravital imaging in mice is a technically challenging and far-from-routine technique. Zebrafish embryos are a common model system for intravital imaging of the metastatic site. However, an adult model system is more likely to recapitulate the events in human patients. We exhibited here that adult *casper* zebrafish provide a simple system for intravital imaging of the metastatic site. We first reported an efficient protocol for the injection of cells into circulation in young adult zebrafish. We then used an intravital imaging protocol to follow individual tumor cells at the metastatic site over the course of 2 weeks. Our results demonstrate that adult *casper* fish are a useful system for performing intravital imaging of the metastatic site. Furthermore, given the low cost of zebrafish and simplicity of our methods, they offer to increase access to intravital imaging.

## Additional files

Additional file 1: Example image of an intravenous injection into the common cardinal vein. (**A**) Example image showing the positioning of the needle and anesthetized zebrafish during intravenous injections. (PDF 3063 kb)

Additional file 2: **Movie S1.** ZMEL1 cells flowing through the sub-dermal vasculature 56 min post-injection. Time code is in seconds. Scale bar is 100 μm. (AVI 8044 kb)

Additional file 3: **Movie S2.** ZMEL1 cells stably arrested in the sub-dermal vasculature 140 min post-injection. Time code is in seconds. Scale bar is 100 μm. (AVI 11701 kb)

Additional file 4: Human tumor cells arrest and extravasate in adult zebrafish but fail to form tumors. (**A**) Images of human melanoma (MDA-MB-435) and breast cancer (LM2) cells that have extravasated in zebrafish 2 days post-injection. Scale bars are 10 μm. (**B**) Images showing the attrition of LM2 cells over time in adult zebrafish following injection. Scale bar is 100 μm. (**C**) Quantification of the fraction of LM2 cells remaining over time in adult zebrafish. *n* = 47 fields in 7 different fish. (**D**) Images of the tails of embryos 1 and 4 DPI (3 and 6 days old) injected with LM2 or MA2 cells. Scale bar is 100 μm. (PDF 1343 kb)

Additional file 5: Table S1. Summary of human cell xenotransplantation experiments. Table summarizing the immunosuppression conditions tested and the results of each method following xenotransplantation. (XLSX 44 kb)

## Abbreviations

AMW: Aquarium makeup water
DPI: Days post-injection
Gy: Gray
H&E: Hematoxylin and Eosin
IV: Intravenous
PBS: Phosphate-buffered saline
RO: Retro-orbital
