# Supplementary figures and images for: Intravital imaging of metastasis in adult Zebrafish
Source: BMC Cancer. 2017 Sep 25;17:660. doi: 10.1186/s12885-017-3647-0 (PMC5613480; doi:10.1186/s12885-017-3647-0)

A

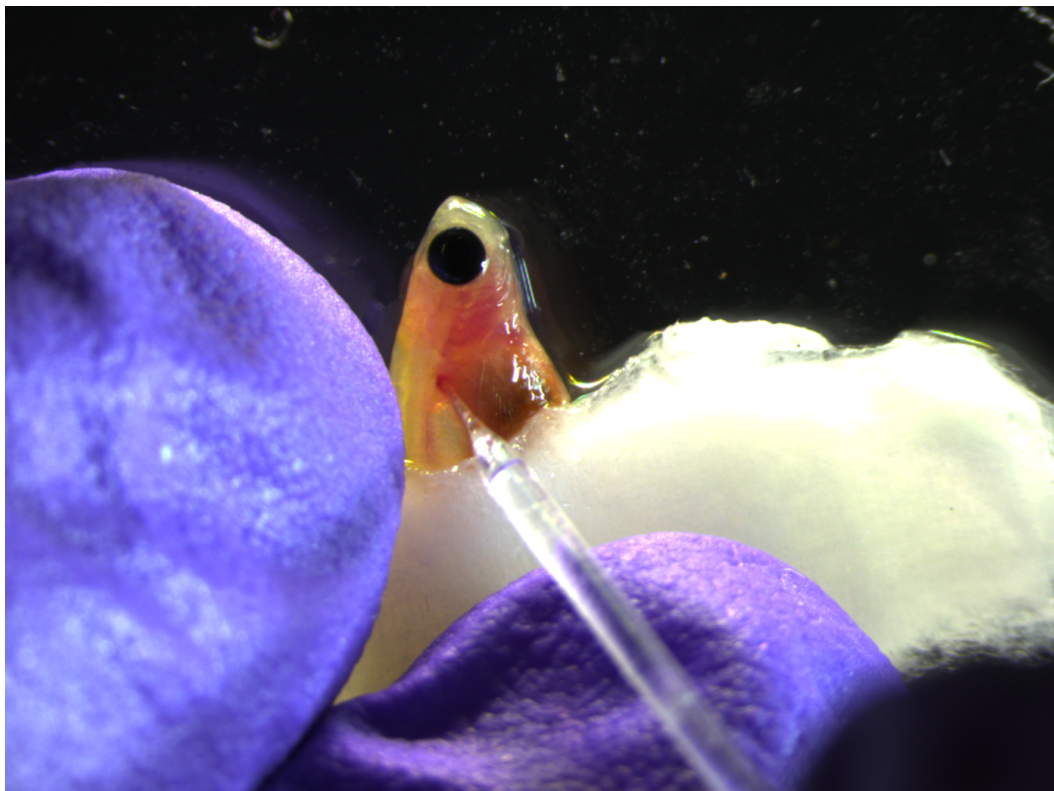

Supplement: Supplementary file 1 — Example image of an intravenous injection into the common cardinal vein. (A) Example image showing the positioning of the needle and anesthetized zebrafish during intravenous injections. (PDF 3063 kb) [file 12885_2017_3647_MOESM1_ESM.pdf]

**A**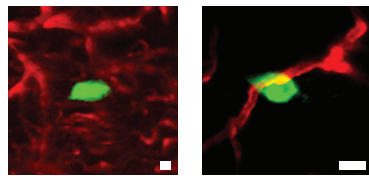**MDA-MB-435****LM2****B**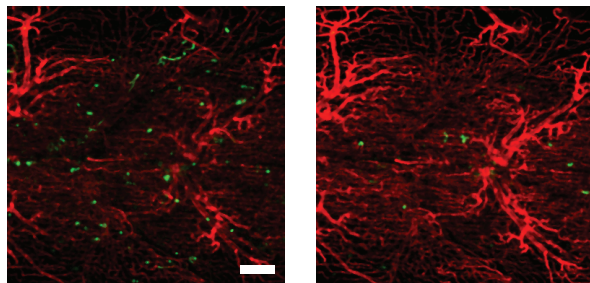**Day 0****Day 1****C**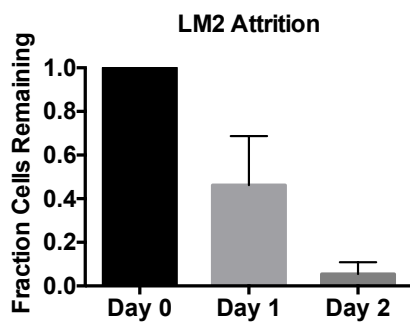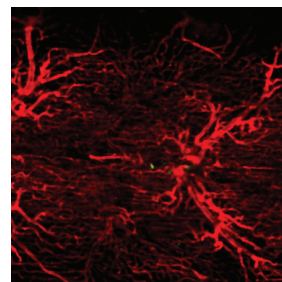**Day 2****D**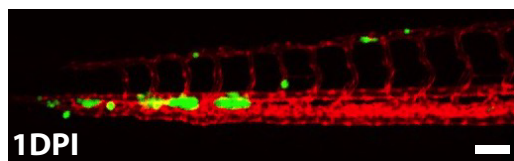**1DPI**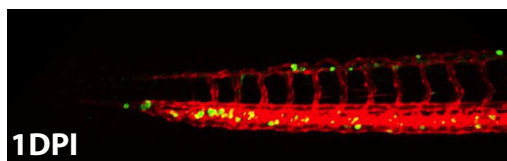**1DPI**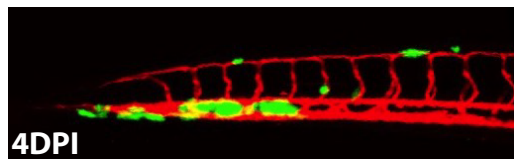**4DPI****MA2**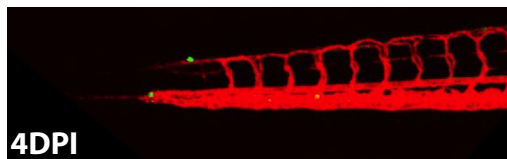**4DPI****LM2**

Supplement: Supplementary file 4 — Human tumor cells arrest and extravasate in adult zebrafish but fail to form tumors. (A) Images of human melanoma (MDA-MB-435) and breast cancer (LM2) cells that have extravasated in zebrafish 2 days post-injection. Scale bars are 10 μm. (B) Images showing the attrition of LM2 cells over time in adult zebrafish following injection. Scale bar is 100 μm. (C) Quantification of the fraction of LM2 cells remaining over time in adult zebrafish. n = 47 fields in 7 different fish. (D) Images of the tails of embryos 1 and 4 DPI (3 and 6 days old) injected with LM2 or MA2 cells. Scale bar is 100 μm. (PDF 1343 kb) [file 12885_2017_3647_MOESM4_ESM.pdf]
